# Competitive Relationship Between *Cleistocalyx operculatus* and *Syzygium jambos* Under Well-Watered Conditions Transforms Into a Mutualistic Relationship Under Waterlogging Stress

**DOI:** 10.3389/fpls.2022.869418

**Published:** 2022-06-10

**Authors:** Fan Yang, Juan Zhang, El-Hadji Malick Cisse, Da-Dong Li, Lu-Yao Guo, Li-Shan Xiang, Ling-Feng Miao

**Affiliations:** ^1^School of Ecological and Environmental Sciences, Hainan University, Haikou, China; ^2^Key Laboratory of Agro-Forestry Environmental Processes and Ecological Regulation of Hainan Province, Center for Eco-Environmental Restoration Engineering of Hainan Province, Haikou, China; ^3^School of Life Science, Hainan University, Haikou, China; ^4^School of Plant Protection, Hainan University, Haikou, China

**Keywords:** adventitious roots, endogenous hormone balance, interspecific relationship, mixed planting pattern, pure planting pattern, redox equilibration

## Abstract

Competition and abiotic stress such as waterlogging (WL) represent main factors limiting plant growth and determining plant resistance and distribution patterns in wetland ecosystems. One of the basic steps for wetland restoration is to plant trees to ensure a quicker recovery and prevent erosion. Plant survival and adaptation are considered criteria of principal priority for the screening of plant species for wetland ecosystem restoration. WL influences plant species in wetlands *via* the deterioration of the plant root environment which leads to oxygen deficiency that affects plant growth, photosynthesis, respiration, and other metabolic processes. A suitable plant species was determined according to tolerance to WL during wetland vegetation recovery activities. Thus, two tree species (*Cleistocalyx operculatus* and *Syzygium jambos*) that showed a certain waterlogging tolerance were chosen to study their behaviors in different planting model and environmental conditions. Given that interspecific relationship should be considered during plant community construction, the eco-physiological adaptable mechanisms between these woody plants under well-watered condition and waterlogging stress were explored. Results showed that both species were waterlogging-tolerant species due to their ability to adapt to submergence conditions for 120 days. Moreover, *C. operculatus* possessed stronger tolerance to waterlogging stress because of a significant adventitious roots biomass accumulation. A competitive relationship was found between *C. operculatus* and *S. jambos* under well-watered condition, and *C. operculatus* showed better growth performance benefited from morphological responses (plant height, number of blade and leaf area) and endogenous hormone variations. In comparison, *S. jambos* suffered some negative effects when the well-watered mixed planting was used. However, the competitive relationship under well-watered condition was transformed into mutualistic relationship under waterlogging stress. The mixed planting under waterlogging condition significantly improved the tolerance of *C. operculatus* and *S. jambos* to waterlogging stress, compared with the monoculture., Especially, *S. jambos* showed improvements in root length, root surface area, and redox equilibration between lower levels of relative conductivity, malondialdehyde, and $O_{2}^{\cdot -}$ and had increased levels of non-enzymatic antioxidant components, such as reduced glutathione and soluble proteins. The interspecific relationship between *C. operculatus* and *S. jambos* was altered by waterlogging stress, and both showed improved tolerance to waterlogging stress. This study can provide a glimmer of light on suitable plant species selection and plant community construction during the revegetation activities in wetland zones. *C. operculatus* and *S. jambos* represent potential candidates in wetland restoration in a mixed planting model.

## Highlights

– *Cleistocalyx operculatus* with better growth performance benefits from water-watered mixed planting pattern.
*– C. operculatus* possessed stronger tolerance to waterlogging stress than *Syzygium jambos*.
– Plant competitive interactions under well-watered conditions become mutualistic under waterlogged conditions; *S. jambos* which has a low tolerance to waterlogging stress seems to gain more benefits.
– The results of this study showed a promising perspective to use *C. operculatus* and *S. jambos* in the restoration of areas that are subject constantly to submergence conditions.

## Introduction

In riparian and wetland zones, waterlogging is the most important environmental stress, which greatly limits the growth, distribution, and survival of terrestrial plants (Miao et al., 2017). This is because the root systems of terrestrial plants in wetland zones suffer from oxygen deficiency in water-saturated soil (Kreuzwieser and Rennenberg, 2014; Li et al., 2022). Thus, many metabolic processes for satisfying the oxygen demand are seriously inhibited, aerobic respiration is transformed into anaerobic respiration, and many enzymes involved in the glycolysis pathway are activated (Yin et al., 2009; Yang et al., 2015). These effects not only affect the absorption and availability of nutrients and water but also limit energy production and stimulate reactive oxygen species (ROS) formation. Indeed ROS are crucial toxic and regulatory molecules in plant which play a main role in plant stress tolerance responses. They are responsible for the oxidative stress caused by their over-accumulation in plant under stress such as waterlogging. Furthermore, ROS are able to catalyze cations signaling events involved in plant stress tolerance (Demidchik, 2015). Metabolic processes are considerably altered by changes in membrane lipid peroxidation, pigments, proteins, nucleic acids, osmoregulation substance, enzyme activity, and hormone levels, which will finally damage the whole growth performance, even to death. However, waterlogging-tolerant species generally have specific adaptable strategies to avoid or tolerate the negative effects of waterlogging, such as the formation and development of aerenchyma tissues and adventitious roots and other physiological adjustments, including the antioxidant machinery that are responsible to scavenge the excessive ROS production during the stress (Seago et al., 2005; Li et al., 2022). Indeed, the antioxidant system plays a crucial role in the detoxification of ROSs in wetland plants and includes both non-enzymatic components and enzymatic components. The non-enzymatic component is composed by molecules such as ascorbic acid (ASA), reduced glutathione (GSH), and proline. Meanwhile the antioxidant enzymatic system is constitute by enzymes such as peroxidase (POD), catalase (CAT), and superoxide dismutase (SOD; Yang et al., 2015). Antioxidant enzymes, such as POD, CAT and SOD, are activated by plants to control ROS production under waterlogging stress (Yang et al., 2015; Seymen, 2021). The over-production of ROS in plant under stress regulated by antioxidants involves also the plant hormone. In fact, ROS represent important second messengers in hormone signaling that play a massive part in the regulation of plant development and stress tolerance (Xia et al., 2015). Furthermore, the hormonal hierarchy of abscisic acid (ABA), indole-acetic acid (IAA), and gibberellin A3 (GA_3_) is involved in the regulation of waterlogging tolerance (Li et al., 2022), and methyl jasmonate (JA-Me) is associated with plant leaf senescence and plant caducity and promotes ethylene production (Voesenek and Bailey-Serres, 2015). The physiological adjustments including the regulation of ROS and the involvement of plant hormones are crucial for the adaptations of plants to environmental stress like WL. Indeed, the tolerance of plant species to WL depends on their capacity to change their morphological and physiological traits (Bertolde et al., 2010). But, also it has been proven that positive plant–plant interactions such as facilitation are very helpful for some species under stressful conditions and such positive interactions usually co-occur with competition (Zhang and Tielbörger, 2019). Thus, it is clear that the adaptation of plant to submergence conditions depends on intern (i.e., physiological processes) and extern factors (i.e., interspecific relation with others species).

Interspecific relationship (competition or facilitation) is an important biotic factor affecting plant species distribution, population dynamics, community construction, and ecosystem services (Lin et al., 2012). It has been reported that the evaluation (experimentally) of the impact of environmental stress on interaction strengths can significantly participate to understand the crucial importance of these two processes (competition or facilitation) in structuring plant communities (Callaway and Walker, 1997; Paquette and Hargreaves, 2021). Competitive relationship indicates the ability of adjacent plants to obtain and use natural resources, such as nutrients, light, and water. For instance, a competitive interaction between adjacent plants occurs when they are competing for limited resources such as soil nutrients that can affect particularly their growth and structure (Xiao et al., 2009). In another hand, facilitation is known as positive interactions among organisms in communities and it has been considered as the primary driving biotic mechanism of plant community succession. It can increase species richness in a large range of environmental conditions (Brooker et al., 2008; Xiao et al., 2009; Michalet and Pugnaire, 2016). Competition and facilitation usually occur simultaneously under environmental stress, and the final neighboring relationship is the net effect of these relationships (Gersani et al., 2001; Mommer et al., 2010). Therefore, the neighboring relationship between plants is variable, and they still mainly use resources for root growth when growth environment resources are insufficient. A previous study showed that morphological and physiological responses to waterlogging stress depend on the comprehensive influences of different abiotic stress stresses, such as the severity, duration, and level (depth) of waterlogging stress, and biotic factors, including life history strategies that enable survival in a highly disturbed environment and neighboring relationship (Vreugdenhil et al., 2006). Moreover, it has been demonstrated that interspecific neighbors relationship depends on abiotic stress (waterlogging) gradient (Grime, 1973; Brose and Tielbörger, 2005). Brose and Tielbörger (2005) reported that abiotic and biotic factors interact in determining plant abundance and distribution in wetlands under submergence conditions. These finding is consistent with the stress gradient hypothesis proposed by Bertness and Callaway (1994).

Although there are many studies that focused on the physiological and morphological adaptations of plant species commonly used in restoration practices for individuals grown in monoculture, there is still a poor understanding of how species interactions may induce competitive or facultative interactions that ultimately modify the environmental conditions that individual plants are growing in despite the anaerobic conditions associated with waterlogging in wetlands. This is of particular importance in different regions threatened by submergence conditions, where two species such *Cleistocalyx operculatus* and *Syzygium jambos* are often used in restoration and reconstruction of riparian and wetland areas. Indeed, *Cleistocalyx operculatus* and *S. jambos* belong to the Myrtaceae family. They are evergreen medicinal woody plants with waterlogging tolerance and often used in the restoration and reconstruction of riparian zones of tropical and subtropical regions (Jing et al., 2001; Sharma et al., 2013; Nguyen et al., 2017; Ma et al., 2019). In the present study, the pure and mixed planting patterns of *C. operculatus* and *S. jambos* under well-watered condition and waterlogging stress were designed and used in answering the following questions: (1) Can these two species benefit from the mixed planting pattern under well-watered conditions? (2) Whether the woody plant with strong waterlogging tolerance gain more benefits than a plant species which possesses a less waterlogging tolerance when they are subjected to waterlogging in a mixed planting? (3) Whether waterlogging environments alter neighboring relationships between two species and whether competition or facilitation is preferred? Thus, changes in interspecific relationships between *C. operculatus* and *S. jambos* under well-watered and waterlogging conditions were explored through physiological analysis based on phenotypic traits, photosynthetic performance, lipid peroxidation, non-enzymatic antioxidant components, antioxidant enzymatic activities, and endogenous hormones.

## Materials and Methods

### Experimental Designs

Two 6-month-old tree species seedlings of *C. operculatus* and *S. jambos* from Zengcheng, Guangdong Province were planted in pairs in a 43 cm × 19 cm × 14 cm (L × W × H) plastic bar-shaped basin, which was filled with red soil, sand, and coconut coir in 4:2:1 (v:v:v) ratio. The chemical and physical properties of the red soil were: pH = 6.23, 33.65 g.kg^−1^ of organic carbon, 58.01 g.kg^−1^ of organic matter, 1.77 g.kg^−1^ of total nitrogen, 0.64 g.kg^−1^ of total phosphorus. Before treatment, fertilizer solution was applied two times at 300 ml in each pot (day 1 and after 2 weeks from March 28, 2019). The experiment was carried out in a greenhouse at Hainan University (20° 03′ 33.2″ N, 110° 20′ 16.9″ E). Healthy plants were selected for a randomized design with treatments consisting of combinations of two irrigation levels (well-watered and waterlogging conditions), and three different planting systems (monoculture of *C. operculatus* or *S. jambos*, and mixed planting between *C. operculatus* and *S. jambos*, two seedlings in each pot) were arranged. Each treatment had at least 24 replicates, which implied at least 144 pots for the whole experiment. The waterlogging depth was 10 cm above the soil surface. For each species, the well-watered and waterlogging treatments were marked as CK and W, respectively, in the monoculture, and marked as CK-M and W-M, respectively, in the mixed planting. For the well-watered treatment, pots were watered daily (100% field capacity). All the experiments were arranged in a complete randomized design. The morphological and physiological assays were determined after 120 days.

### Morphological Parameters

To determine the increments in plant height (IPH) and blade number (IBN), the plant height and number of blades were measured at the beginning (day 1) and the end (day 120) of treatments. The IPH was calculated as Height_120_-Height_1_, and IBN was calculated as N_120_-N_1_. The leaf area (LA) was determined before the end of the experiment with a portable area meter (LI-3000C; Li-COR, Lincoln, United States; Pu et al., 2021) in 3 leaves for each pot and 8 pots for each treatment. The fresh weights (direct index of plant growth) of adventitious roots, original roots, underground biomass, and aboveground biomass were measured immediately after harvesting. The roots were scanned with a scanner (Epson v750, China), and root length and root surface area were determined with a WinRHIZO analysis system (Li et al., 2022).

### Photosynthesis-Related Parameters

The young fresh leaves were collected at the end of the treatment. Chlorophyll a (Chl a), chlorophyll b (Chl b), total chlorophyll, and carotenoids (Caro) were quantified after extraction in 95% (v/v) ethanol with a spectrophotometer (6,800 PC, Shanghai Metrum Instrument Co., Ltd) at 470, 640, and 665 nm. The net photosynthetic rate (Pn), intercellular carbon dioxide (Ci), stomatal conductance (Gs), and transpiration rate (Tr) were determined once at the end of the treatment from 08:30 to 11:30 h using a 3051D photosynthetic device (Zhejiang Top Instruments Co., Ltd.) according to the methods of Li et al. (2022). Fv/Fm (maximum photochemical quantum yield of PSII) was measured with a Junior-PAM chlorophyll fluorometer (Walz, Effeltrich, Germany) according the method of Liu et al. (2020).

### Relative Conductivity, Malondialdehyde, and Superoxide Anion ( $O_{2}^{\cdot -}$ ) Contents

Relative conductivity was measured according to the method described by Fan et al. (2015) with some modifications. Fresh leaves were cleaned with deionized water, and 10 discs from the fresh leaves were incubated with 15 ml of deionized water. Initial conductivity (C1) was recorded after 2 h, and then the samples were boiled for 45 min, and then the final conductivity C2 was measured. RC was calculated with the following formula: relative conductivity (%) = C1/C2 × 100.

Malondialdehyde (MDA) was determined according to the method of Yang and Miao (2010). Fresh samples were homogenized with 0.1% trichloroacetic acid (TCA) and then centrifuged at 11000 × g for 8 min. The supernatant (1 ml) was mixed with 4 ml of 20% TCA solution containing 0.5% thiobarbituric acid and incubated at 100°C for 30 min. The reaction mixture was cooled on an ice bath and then centrifuged at 11,000 × g for 8 min. Absorbance was measured first at 530 nm and corrected at 600 nm.

The production rate of $O_{2}^{\cdot -}$ was quantified using the modified method described by Yang et al. (2011). Fresh samples (0.15 g) were homogenized in 2 ml of phosphate buffer (pH 7.8) and then centrifuged at 10000 × g for 15 min. The supernatant was decanted and mixed with phosphate buffer. The reaction mixture included 1 ml of supernatant, 0.75 ml of 65 mM phosphate buffer, and 0.25 ml of 10 mM hydroxylamine hydrochloride. The mixture was incubated at 25°C for 20 min and then added to 1 ml of 17 mM sulfanilamide and 1 ml of 7 mM α-naphthylamine. The mixture was kept at 30°C for 30 min and then mixed with 4 ml of trichloromethane before centrifugation at 10,000 × g at 4°C for 3 min. Absorbance was measured at 530 nm, and NaNO_2_ was used as the standard curve for the calculation of the $O_{2}^{\cdot -}$ content.

### Non-enzymatic Antioxidant Matter Determination

The concentration of proline was measured with the method of Bates et al. (1973) and Yang and Miao (2010) with some modifications. Approximately 200 mg of fresh leaf samples was mixed with 5 ml of 3% sulfosalicylic acid. An acid–acetic ninhydrin solution was used as the reagent. Then, 1 ml of an acid–acetic ninhydrin reagent and 1 ml of glacial acetic acid were successively added to 1 ml of the homogenate. The mixture was then incubated at 100°C for 1 h. The reaction was stopped by cooling the samples on ice water. Toluene was used in extracting the chromophore-containing phase, and absorbance was recorded at 520 nm.

Soluble proteins were determined according to the method described by Bradford (1976). Coomassie Brilliant Blue G-250 was used. Approximately 2 ml of phosphate buffer (pH 7.8) was used as the extraction solution, and 100 mg of fresh samples was mixed with phosphate buffer. Absorbance was read at 595 nm, and protein concentration was determined with a standard curve.

Reduced GSH (reduced glutathione) and ASA (ascorbic acid) levels were measured according to the manufacturer’s instructions with the method of Yang et al. (2015) and Anee et al. (2019). Approximately 100 mg of fresh leaves was ground in liquid nitrogen and homogenized with PBS (5 mg of fresh leaves in 50 μl of PBS). The mixture was centrifuged at 10,000 rpm for 30 min at 4°C. The assay was performed with kits provided by Nanjing Jiancheng Bioengineering Institute (China).

### Antioxidant Enzymatic Activity Determination

Fresh leaf samples (0.2 g) were ground and homogenized with tissue grinder instrument scientific laboratory homogenizer equipment (JXFSTPRP-24, Shanghai Jingxin Industrial Development Co., Ltd., China). The mixture contained liquid nitrogen, 5 ml of 50 mM sodium phosphate buffer (pH 7.8), and 1% polyvinylpolypyrrolidone. The mixture was centrifuged at 10,000 rpm, 4°C for 15 min, and the supernatant was used to perform for the antioxidant enzymatic activities determination.

Expressed as unit· g^−1^·FW·min^−1^, POD (peroxidase) activity was determined with the method described Yang and Miao (2010) according to absorption change caused by guaiacol oxidation at 470 nm. SOD (superoxide dismutase) activity was measured using the method described by Yang and Miao (2010) according to the inhibition of nitroblue tetrazolium chloride reduction. Absorbance was recorded at 560 nm with a spectrophotometer (UV-1800PC, Shanghai Mapada Instruments Co., Ltd., China). CAT (catalase) was determined in accordance with the manufacturer’s instructions with the method described by Yang et al. (2015). Phosphate buffer was provided with the assay kit from Nanjing Jiancheng Bioengineering Institute, China.

### Endogenous Hormone Parameters

Endogenous IAA, ABA, GA_3_, and JA-Me were extracted and purified with the methods described by Bollmark et al. (1988). Hormones were quantified through ELISA with the methods described as Yang et al. (2001). The mouse monoclonal antigens and antibodies against IAA, ABA, GA_3_, and JA-Me horseradish peroxidase used in ELISA were produced at the Phytohormones Research Institute, and fresh samples were sent for determination in China Agricultural University.

### Statistical Analyses

Experimental data processing statistics, analysis, and mapping were performed using SPSS 23.0, Canoco 5.0 and GraphPad Prism 9. All data were tested for normal distribution and homogeneity of variance before analysis. One-way analysis of variance followed by Tukey’s multiple comparison tests was used in establishing significant differences between pure planting and mixed planting under waterlogging stress at 1 and 5% levels of significance. Correlation analysis was performed with R software (R Development Core Team, 2019, version 3.6.1). PCA was used in assessing and comparing the important parameters of monoculture and mixed planting on *C. operculatus* and *S. jambos* under waterlogging conditions.

## Results

### Effects on Morphological Characteristics

*Cleistocalyx operculatus* had better growth rates and performance (IPH, LA, IBN and fresh biomass accumulation) under well-watered conditions compared to *S. jambos*. All waterlogging-treated seedlings from *C. operculatus* and *S. jambos* survived after 120 days of waterlogging treatment, but their growth rates significantly varied from each other. In *C. operculatus*, compared with CK treatment, CK-M treatment significantly increased the IPH and LA (*p* < 0.05), whereas non-significant differences in the IBN were found between the CK and CK-M treatments (Table 1). However, in *S. jambos* CK-M had only increased significantly the IBN compared to the control. Waterlogging significantly increased plant height and leaf area in the monoculture group of *C. operculatus* compared to the CK group, whereas non-significant differences in IPH, IBN, and LA were found between W and W-M treatments (*p* < 0.05). In *S. jambos*, compared with CK treatment, W treatment significantly decreased the IPH, IBN, and LA in the group subjected to the monoculture. W treatment also significantly decreased IBN and LA in the mixed planting, but the W-M treatment significantly increased the IPH relative to the CK-M treatment (*p* < 0.05; Table 1).

**Table 1:** The variations of IPH (increment in plant height), IBN (increment in number of blades), and LA (leaf area) in *Cylindrocopturus operculatus* and *Syzygium jambos* among different treatments.

Species	Treatments	IPH (cm)	IBN (piece)	LA (cm^2^)
*C. operculatus*	CK	13.8 ± 0.87 b^**^	20.4 ± 1.60 a^**^	333.63 ± 14.12 c^**^
	CK-M	22.3 ± 0.26 a^**^	22.8 ± 0.80 a^**^	619.93 ± 37.68 a^**^
	W	23.9 ± 0.24 a^**^	21.8 ± 0.20 a^**^	425.41 ± 39.08 b^**^
	W-M	22.6 ± 0.40 a^**^	21.4 ± 0.25 a^**^	509.34 ± 17.79 b^**^
*S. jambos*	CK	8.2 ± 0.46 B	5.2 ± 0.58 C	214.0 ± 14.32 AB
	CK-M	8 ± 0.27 B	16.2 ± 0.20 A	234.8 ± 59.35 A
	W	4.5 ± 0.22 C	4.0 ± 0.0 D	119.5 ± 7.91 C
	W-M	10.8 ± 0.30 A	11.8 ± 0.37 B	174.2 ± 3.30 B

Values are means ± SE (*n* = 6). Different little and capital letters indicate significant differences among different treatments within the same species according to ANOVA’s test (*p* < 0.05). CK, pure planting pattern under well-watered condition; CK-M, mixed planting pattern under well-watered condition; W, pure planting pattern under waterlogging condition, W-M; mixed planting pattern under waterlogging condition. The signs such as ^**^ represent the significant difference between the two species under the same treatment at *p* < 0.05 and *p* < 0.01; ns, means non-significant.

### Effects on Biomass Characteristics

**Figure 1:**
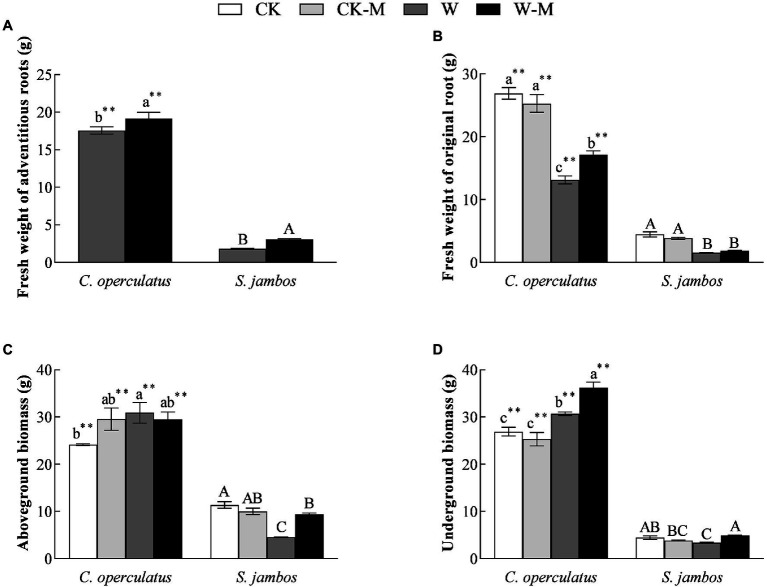
The variations of fresh weight of adventitious roots **(A)**, fresh weight of original root **(B)**, aboveground biomass **(C)** and underground biomass **(D)** in *Cylindrocopturus operculatus* and *Syzygium jambos* among different treatments. Values are means ± SE (*n* = 6). Different little and capital letters indicate significant differences among different treatments within the same species according to ANOVA’s test (*p* < 0.05). CK, pure planting pattern under well-watered condition; CK-M, mixed planting pattern under well-watered condition; W, pure planting pattern under waterlogging condition, W-M; mixed planting pattern under waterlogging condition. The sign ^**^ concerns the significance difference between the two species under the same treatment. ^**^p < 0.01.

*Cleistocalyx operculatus* showed a massive accumulation of the fresh weight from adventitious and original roots, aboveground and belowground biomass compared to *S. Jambos* under well-watered conditions. Moreover, results showed that no significant differences in the fresh weights of original roots, aboveground and underground biomass were found between the CK and CK-M treatments in both species (Figure 1). As shown in Figure 1A, W-M treatment significantly increased the fresh weights of the adventitious roots of both species, compared with the W treatment, and the fresh weights of the adventitious roots of *C. operculatus* were significantly higher than those of *S. jambos* (*p* < 0.05) under the W-M treatment. Interestingly, the W treatment significantly increased the aboveground biomass and underground biomass of *C. operculatus* in the monoculture group, whereas *S. jambos* showed the opposite trends (*p* < 0.05). Compared with the monoculture group, the mixed planting under waterlogging conditions increased significantly the aboveground and underground biomass of *S. jambos* but only increased the underground biomass of *C. operculatus* (*p* < 0.05; Figures 1C,D).

**Figure 2:**
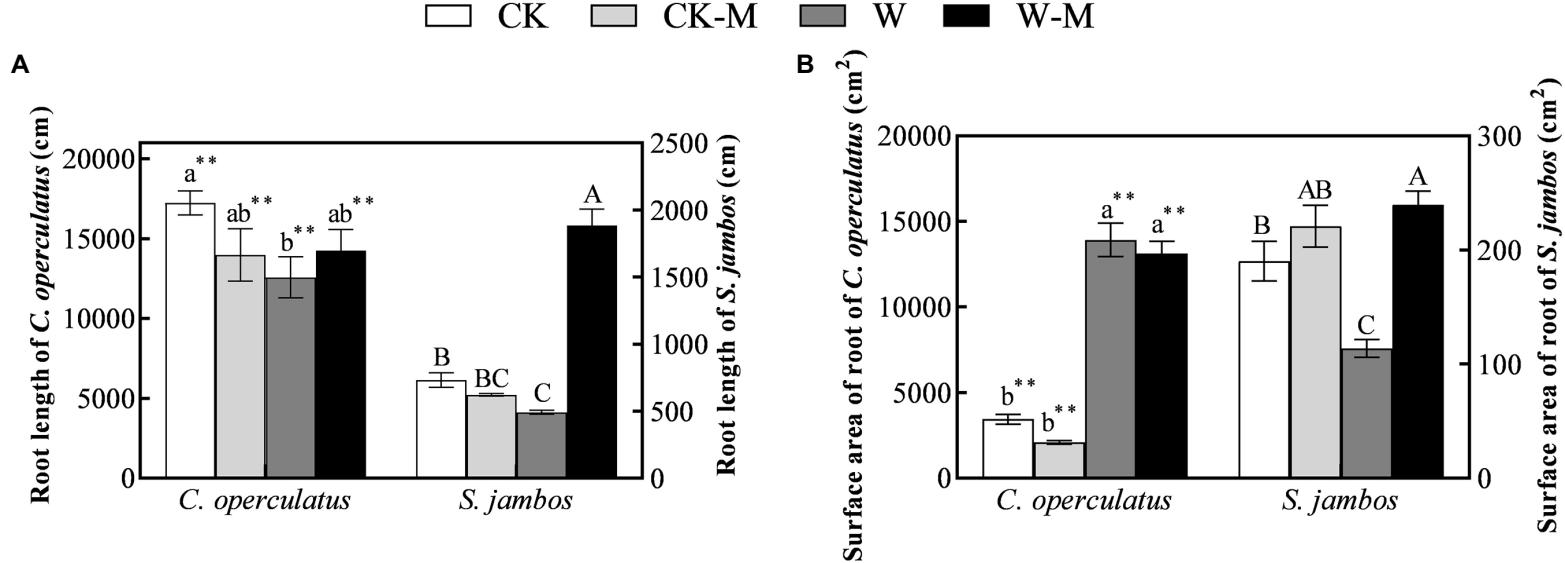
The variations of root length **(A)** and root surface area **(B)** in *C. operculatus* and *S. jambos* among different treatments. Values are means ± SE (*n* = 6). Different little and capital letters indicate significant differences among different treatments within the same species according to ANOVA’s test (*p* < 0.05). CK, pure planting pattern under well-watered condition; CK-M, mixed planting pattern under well-watered condition; W, pure planting pattern under waterlogging condition, W-M; mixed planting pattern under waterlogging condition. The sign ^**^ concerns the significance difference between the two species under the same treatment. ^**^p < 0.01.

Under well-watered conditions *C. operculatus* displayed a greater root length increment compared to *S. jambos* (Figure 2A). Moreover, the root length and root surface area of *C. operculatus* within the same treatment were significantly higher than those of *S. jambos* (*p* < 0.01). Further, *S. jambos* showed a significant increase of its surface areas of roots compared to *C. operculatus*. The results showed no significant differences in the root lengths and root surface areas of *C. operculatus* and *S. jambos* between the CK and CK-M treatments (Figure 2). The W treatment significantly inhibited increases in the root length and root surface area of *S. jambos*, whereas the W-M treatment alleviated this inhibition, significantly improving these parameters. However, waterlogging stress significantly increased the root surface area in *C. operculatus* in the W and W-M treatments, and the root surface areas in the waterlogging treatments were significantly larger than those in the well-watered treatments. The W treatment significantly inhibited increase in root length in *C. operculatus*, whereas W-M could alleviate this inhibition.

### Effects on Photosynthesis-Related Characteristics

As shown in Table 2, the levels of Chl a, Chl b, and total chlorophyll in the CK-M treatment were significantly higher than those in the CK treatment in *C. operculatus* (*p* < 0.05) but obviously decreased after the W treatment. The W-M treatment did not offset the decreases. In *S. jambos*, the Chl a, Chl b, total chlorophyll, and Caro levels were significantly lower in the CK-M and W treatments than in the CK treatment. The W-M treatment significantly improved these parameters, compared with the W treatment.

**Table 2:** The variations of chlorophyll a, chlorophyll b, total chlorophyll and carotenoids in *C. operculatus* and *S. jambos* among different treatments.

Species	Treatments	Chlorophyll a (μg/g·Fw)	Chlorophyll b (μg/g·Fw)	Total Chl (μg/g·Fw)	Carotenoids (μg/g·Fw)
*C. operculatus*	CK	277.1 ± 5.56 b^**^	42.76 ± 1.79 b^**^	319.85 ± 6.95 b^**^	202.66 ± 6.69 a^**^
	CK-M	304.97 ± 10.94 a^*^	55.05 ± 1.78 a^**^	360.02 ± 12.49 a^ns^	191.24 ± 9.37 ab^**^
	W	206.98 ± 7.92 c^ns^	36.70 ± 3.66 bc^**^	243.68 ± 10.92 c^*^	209.09 ± 5.71 a^**^
	W-M	212.23 ± 5.37 c^**^	30.59 ± 1.68 c^**^	242.82 ± 5.93 c^**^	171.39 ± 7.38 b^**^
*S. jambos*	CK	598.8 ± 13.82 A	124.98 ± 8.64 A	723.78 ± 19.47 A	141.69 ± 7.15 A
	CK-M	269.1 ± 4.9 B	72.04 ± 3.68 B	341.18 ± 5.93 B	84.59 ± 3.54 B
	W	209.50 ± 4.25 C	50.69 ± 3.55 C	260.18 ± 2.00 C	66.67 ± 4.25 C
	W-M	273.53 ± 5.84 B	67.73 ± 4.71 B	341.26 ± 6.84 B	87.23 ± 4.12 B

Values are means ± SE (*n* = 6). Different little and capital letters indicate significant differences among different treatments within the same species according to ANOVA’s test (*p* < 0.05). CK, pure planting pattern under well-watered condition; CK-M, mixed planting pattern under well-watered condition; W, pure planting pattern under waterlogging condition, W-M; mixed planting pattern under waterlogging condition. The signs such as ^*^, ^**^ represent the significant difference between the two species under the same treatment at *p* < 0.05 and *p* < 0.01; ns, means non-significant.

The W treatment significantly decreased the levels of Pn, Gs, and Ci in both species in the monoculture group, and the mixed planting under waterlogging conditions (W-M) increased Pn and Ci in both species (*p* < 0.05; Table 3). The Pn values of *C. operculatus* were significantly higher than those of *S. jambos* in the same treatment (*p* < 0.01), and the values of Gs and Tr in *C. operculatus* were significantly higher than those of *S. jambos* (*p* < 0.01) under mixed planting patterns (CK-M and W-M).

**Table 3:** The variations of Pn, Gs, Tr, and Ci in *C. operculatus* and *S. jambos* among different treatments.

Species	Treatments	Pn (μmol·m^−2^·s^−1^)	Gs (mol·m^−2^·s^−1^)	Tr (mmol·m^−2^·s^−1^)	Ci (μmol·mol^−1^)
*C. operculatus*	CK	6.53 ± 0.05 b^*^	0.13 ± 0.01 b^ns^	3.48 ± 0.34 a^*^	455.78 ± 14.14 a^ns^
	CK-M	7.7 ± 0.05 a^**^	0.14 ± 0.01 ab^**^	3.41 ± 0.13 a^**^	477.2 ± 11.95 a^ns^
	W	4.52 ± 0.05 d^**^	0.07 ± 0.01 c^ns^	2.9 ± 0.04 a^**^	415.54 ± 1.57 b^**^
	W-M	4.91 ± 0.07 c^**^	0.17 ± 0.01 a^**^	3.29 ± 0.19 a^**^	479.28 ± 7.95 a^ns^
*S. jambos*	CK	6.03 ± 0.15 A	0.14 ± 0.01 A	2.41 ± 0.1 A	438.38 ± 14.6 A
	CK-M	5.39 ± 0.07 B	0.06 ± 0.01 B	2.40 ± 0.13 A	467.58 ± 10.51 A
	W	3.03 ± 0.12 D	0.06 ± 0.01 B	1.41 ± 0.25 B	363.02 ± 11.23 B
	W-M	3.4 ± 0.05\u00B0C	0.06 ± 0.01 B	1.54 ± 0.06 B	467.12 ± 10.3 A

Values are means ± SE (*n* = 6). Different little and capital letters indicate significant differences among different treatments within the same species according to ANOVA’s test (*p* < 0.05). CK, pure planting pattern under well-watered condition; CK-M, mixed planting pattern under well-watered condition; W, pure planting pattern under waterlogging condition, W-M; mixed planting pattern under waterlogging condition. The signs such as ^*^, ^**^ represent the significant difference between the two species under the same treatment at *p* < 0.05 and *p* < 0.01; ns, means non-significant.

**Figure 3:**
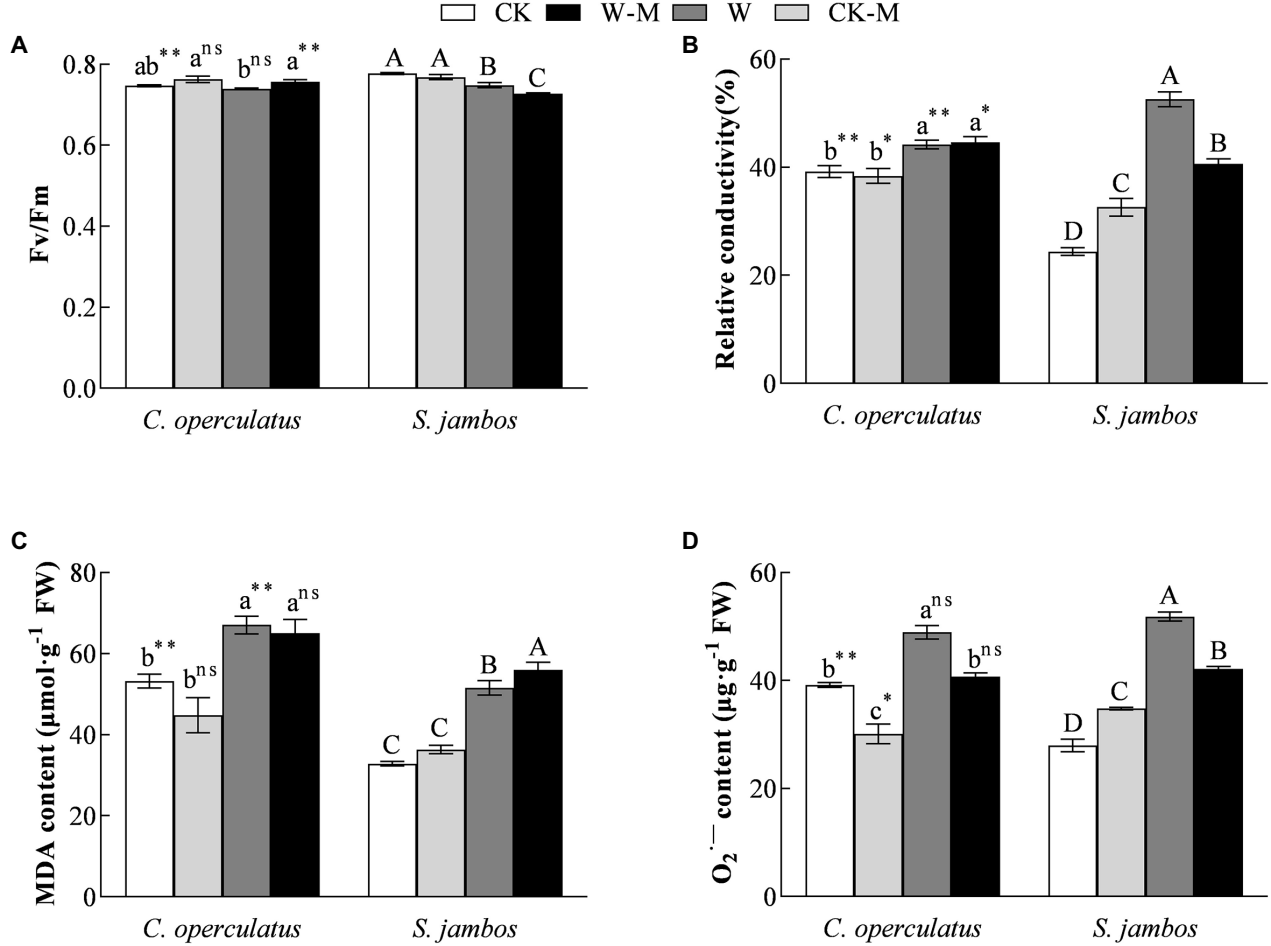
The variations of Fv/Fm (maximum photochemical quantum yield of PSII) **(A)**, relative conductivity **(B)**, MDA content **(C)** and $O_{2}^{\cdot -}$ content **(D)** in *C. operculatus* and *S. jambos* among different treatments. Values are means ± SE (*n* = 6). Different little and capital letters indicate significant differences among different treatments within the same species according to ANOVA’s test (*p* < 0.05). CK, pure planting pattern under well-watered condition; CK-M, mixed planting pattern under well-watered condition; W, pure planting pattern under waterlogging condition, W-M; mixed planting pattern under waterlogging condition. The signs such as ^*^, ^**^, and ns concern the significance difference between the two species under the same treatment. ^*^p < 0.05; ^**^p < 0.01; ns, non-significant.

As shown in Figure 3A, compared with the monoculture group (CK), the mixed planting (CK-M) had no significant effects on Fv/Fm in both species under well water conditions. The W treatment significantly decreased Fv/Fm in *S. jambos* but had no significant effects on *C. operculatus*. Compared with the monoculture group (W), the mixed planting (W-M) significantly improved Fv/Fm in *C. operculatus* and decreased Fv/Fm in *S. jambos* under waterlogging conditions (*p* < 0.01). In addition, significant difference in Fv/Fm was found between *C. operculatus* and *S. jambos* in the CK and W-M treatments (*p* < 0.01).

### Effects on Relative Conductivity, MDA, and $O_{2}^{\cdot -}$ Contents

**Figure 4:**
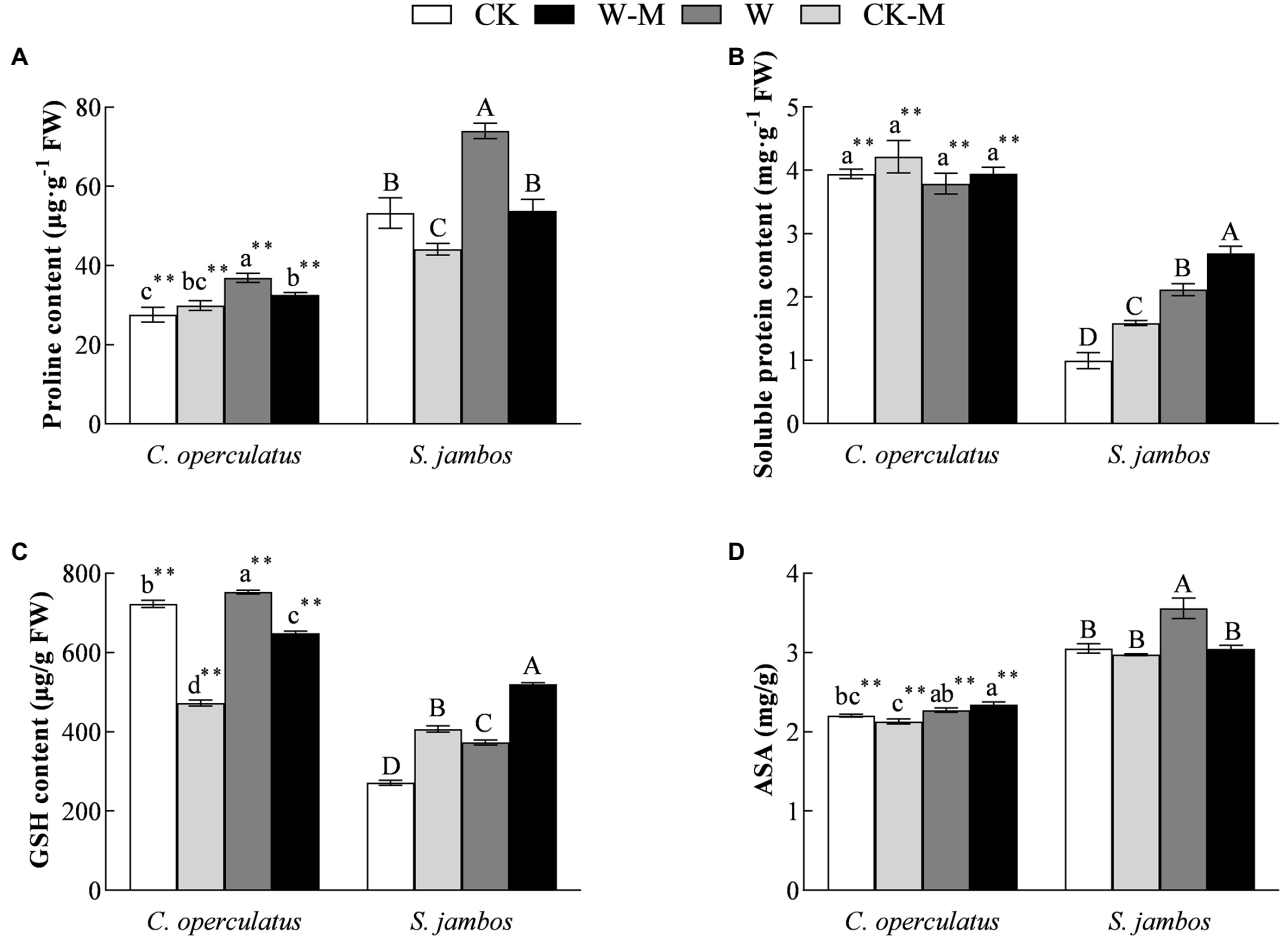
The variations of proline **(A)**, soluble protein content **(B)**, GSH content **(C)** and ASA **(D)** in *C. operculatus* and *S. jambos* among different treatments. Values are means ± SE (*n* = 6). Different little and capital letters indicate significant differences among different treatments within the same species according to ANOVA’s test (*p* < 0.05). CK, pure planting pattern under well-watered condition; CK-M, mixed planting pattern under well-watered condition; W, pure planting pattern under waterlogging condition, W-M; mixed planting pattern under waterlogging condition. The sign ^**^ concerns the significance difference between the two species under the same treatment. ^**^p < 0.01.

Compared with the CK treatment, the W treatment significantly increased relative conductivity (Figure 3B), MDA content (Figure 3C), and $O_{2}^{\cdot -}$ content (Figure 4D) in both species. The mixed planting under well water conditions (CK-M) significantly decreased the $O_{2}^{\cdot -}$ level of *C. operculatus* and significantly increased relative conductivity and $O_{2}^{\cdot -}$ level in *S. jambos*, compared with the monoculture (CK). Compared with the monoculture (W), the mixed planting under waterlogging conditions (W-M) significantly inhibited the accumulation of $O_{2}^{\cdot -}$ in both species. In addition, compared with the W treatment, the W-M treatment significantly reduced the relative conductivity in *S. jambos* (*p* < 0.05) and had insignificant effects on relative conductivity and MDA in *C. operculatus* (*p* ≥ 0.05).

### Effects on Proline, Soluble Protein, GSH, and ASA Contents

Compared with the CK treatment, the W treatment significantly increased proline, soluble protein, GSH, and ASA content in *S. jambos* (Figures 4A–D) and significantly increased the content of proline and GSH in *C. operculatus.* The mixed planting under well water conditions (CK-M) significantly decreased proline content in both species and GSH content in *C. operculatus* and significantly increased the content of soluble protein and GSH in *S. jambos*, compared with the monoculture (CK). Compared with the monoculture (W), the mixed planting under waterlogging conditions (W-M) significantly decreased proline content in both species, GSH content in *C. operculatus*, and ASA in *S. jambos*. The W-M treatment significantly increased the content of soluble protein and GSH in *S. jambos*. In addition, compared with the W treatment, the W-M treatment significantly reduced relative conductivity in *S. jambos* (*p* < 0.05).

### Effects on Antioxidant Enzymatic Activities

**Figure 5:**
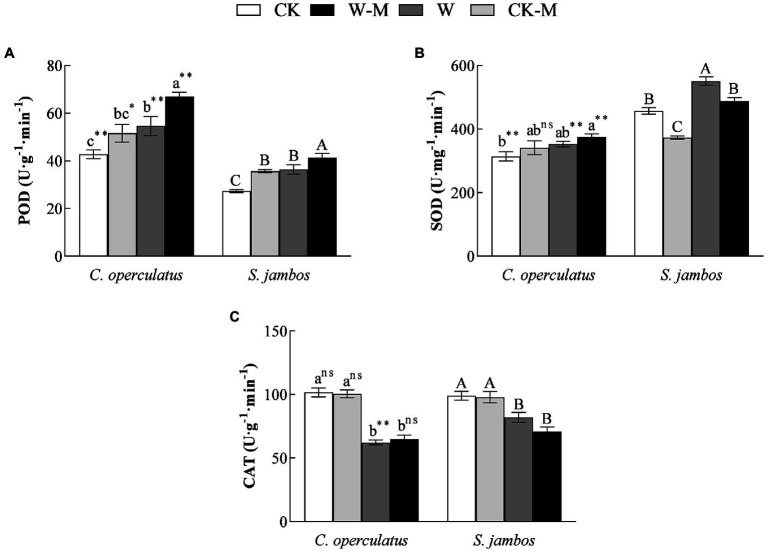
The variations of POD (peroxidase, **A**), SOD (superoxide dismutase, **B**), and CAT (catalase, **C**) in *C. operculatus* and *S. jambos* among different treatments. Values are means ± SE (*n* = 6). Different little and capital letters indicate significant differences among different treatments within the same species according to ANOVA’s test (*p* < 0.05). CK, pure planting pattern under well-watered condition; CK-M, mixed planting pattern under well-watered condition; W, pure planting pattern under waterlogging condition, W-M; mixed planting pattern under waterlogging condition. The signs such as ^*^, ^**^, and ns concern the significance difference between the two species under the same treatment. ^*^p < 0.05; ^**^p < 0.01; ns, no-significant.

The mixed planting under well water conditions (CK-M) significantly increased POD activities in both species, significantly decreased SOD activities in *S. jambos*, and had effects on CAT activities in both species, compared with the monoculture (CK; Figure 5). Compared with the CK treatment, the W treatment significantly increased POD activities (Figure 5A) in both species and SOD activities (Figure 5B) in *S. jambos* but significantly decreased CAT activities (Figure 5C) in both species. Compared with the monoculture (W), the mixed planting under waterlogging conditions (W-M) significantly increased POD activities in both species, decreased SOD activities in *S. jambos*, and had effects on CAT activities in both species. In addition, POD activity in *C. operculatus* was significantly higher than that in *S. jambos* within the same treatment (*p* < 0.05). The mixed planting resulted in a slightly higher increase in SOD activity in *C. operculatus* than the monoculture under well-watered or waterlogging conditions and significantly decreased SOD activity in *S. jambos* (Figure 5; *p* < 0.05).

### Effects on Endogenous Hormones Contents

**Figure 6:**
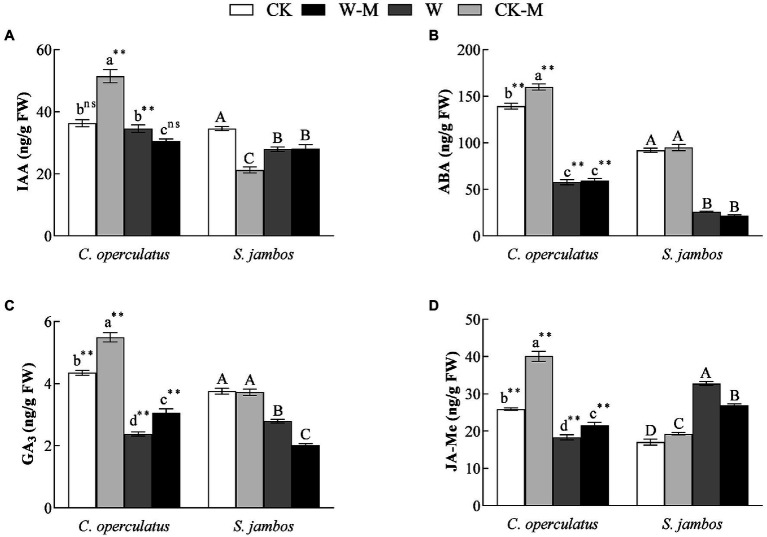
The variations of IAA **(A)**, ABA **(B)**, GA_3_ **(C)**, and JA-Me **(D)** in *Cylindrocopturus operculatus* and *Syzygium jambos* among different treatments. Values are means ± SE (*n* = 6). Different little and capital letters indicate significant differences among different treatments within the same species according to ANOVA’s test (*p* < 0.05). CK, pure planting pattern under well-watered condition; CK-M, mixed planting pattern under well-watered condition; W, pure planting pattern under waterlogging condition, W-M; mixed planting pattern under waterlogging condition. The signs such as ^**^ and ns concern the significance difference between the two species under the same treatment. ^**^p < 0.01; ns, no-significant.

Compared with the monoculture (CK), the mixed planting under well water conditions (CK-M) significantly increased the levels of IAA (Figure 6A), ABA (Figure 6B), GA_3_ (Figure 6C), and JA-Me (Figure 6D) in the leaves of *C. operculatus*, and significantly increased JA-Me content but significantly decreased IAA content in leaves of *S. jambos*, and had no effects on ABA content in the leaves of *S. jambos*. Compared with the CK treatment, the W treatment significantly decreased ABA and GA_3_ content in both species; meanwhile it decreased significantly IAA content in *S. jambos*, significantly decreased JA-Me content in *C. operculatus*, and significantly increased JA-Me content in *S. jambos.* Compared with the monoculture (W), the mixed planting under waterlogging conditions (W-M) significantly increased the content of GA_3_ and JA-Me in *C. operculatus*, significantly decreased their content in *S. jambos*, significantly decreased IAA content in *C. operculatus*, had no effect on *S. jambos*, and had no effects on ABA content in both species (Figure 6).

### Correlation Analysis, PCA, and Subordinate Function Analysis

The results of PCA showed that the total contribution rates of PC1 and PC2 reached 92.81% in *C. operculatus*. The driving factors included photosynthetic pigment indexes (Chl a, Chl b, and total Chl), Pn, fresh weight of original root biomass, root length, root surface area, MDA, GSH, ASA, $O_{2}^{\cdot -}$ content, relative conductivity, CAT activity, and leaf hormones (ABA, GA_3_, IAA, and JA-Me). The differences among treatment groups were significant and can be easily differentiated and identified (Supplementary Figure 1). The total contribution rates of PC1 and PC2 were 80.89% for *S. jambos* (Supplementary Figure 2). The driving factors included root surface area, adventitious root biomass, original root biomass, photosynthetic pigment indexes (Chl a, Chl b and total Chl), Pn, Tr, Fv/Fm, MDA, GSH, ASA, $O_{2}^{\cdot -}$ content, relative conductivity, and ABA, GA_3_, and JA-Me content.

## Discussion

The results of the present research work showed that *C. operculatus* displayed more improvement in plant growth in the pure planting and monoculture. The mixed planting showed better advantages in plant growth and development for *S. jambos* seedlings under waterlogging stress. Indeed, the increase in morphological traits (IPH, IBN, LA, root length, and root surface area) and the enhancement of biomass accumulation in *C. operculatus* in the CK-M treatment (Table 1 and Figures 1, 2) suggested that *C. operculatus* had good growth performance and received greater benefits than *S. jambos*, which showed poor growth performance and suffered from negative effects when both were species were cultured under well-watered condition. In another words, a competitive relationship existed between *C. operculatus* and *S. jambos* under this condition. Moreover, mixed planting system under stress conditions was significantly benefit for *S. jambos* in term of root length and root surface area, whereas it has no obvious effects in *C. operculatus* seedlings. Also compared to *C. operculatus*, the mixed planting has significantly alleviated damages in photosystem apparatus in *S. jambos* under waterlogging stress. Waterlogging has induced MDA, relative conductivity and superoxide anions production in both species. The soluble protein and GSH content in *C. operculatus* within the same treatment was significantly higher than that of *S. jambos*, whereas proline and ASA content showed an opposite trend. The antioxidant machinery played a major role in both species under stress. Furthermore, the phytohormonal profile was affected significantly by the type of specie, planting system and stress.

The survival of all the tested plants after 120 days of waterlogging suggested that *C. operculatus* and *S. jambos* are waterlogging-tolerant woody tree species, but the morphological and physiological responses to waterlogging of *C. operculatus* were better than those of *S. jambos*. Hence, *C. operculatus* possessed stronger waterlogging tolerance. Long-term waterlogging stress inhibited the growth of original root systems and even caused partial death of the original root system by causing root anoxia (Kaur et al., 2020). Thus, waterlogging stress caused a significant reduction in original root biomass, Pn, and pigment content in both species. Moreover, waterlogging induces the growth and development of adventitious roots at the bases of plant stems in waterlogging-tolerant species; this feature is an important survival and regulation mechanism (Bellini et al., 2014; Li et al., 2022). Adventitious roots can replace the normal functions of original roots that died from severe waterlogging stress and oxygen deficiency.

The competitive or facilitative effects among adjacent plants can be modified by environmental factors or biotic factors. For instance, the relationship between *Phragmites australis* and *Spartina alterniflora* decreased with tidal zone, and the mixed planting promoted the tillering production of the two species (Yuan et al., 2013). Waterlogging can decrease the growth, primary root fresh weight, total plant fresh weight, and total leaf area of *C. operculatus* when it is planted alone in one pot (Li et al., 2022). However, the W treatment increased the IBH levels, root surface area, aboveground biomass, and underground biomass (Table 1; Figures 1, 2) in *C. operculatus* relative to those in the CK treatment when the plants were placed in one pot. This result suggested that the intra-specific relationship of *C. operculatus* further improved waterlogging tolerance. This effect was further enhanced by the abundant adventitious roots. These mechanisms may be the adaptable strategies that *C. operculatus* uses to survive under waterlogging stress. The adventitious roots facilitated O_2_, water, and nutrient uptake (Ayi et al., 2016; Li et al., 2022). *C. operculatus* possesses efficient and positive adaptable strategies for alleviating waterlogging damage and can reform up-vertical adventitious roots from primary roots to adapt to waterlogging stress after the artificial removal of normal adventitious roots (Li et al., 2022).

*Syzygium jambos* suffered from negative effects under the well-watered mixed planting (CK-M), and its growth and development were negatively affected by in contrast to *C. operculatus* in the pure planting (CK). Nevertheless, the mixed planting under waterlogging conditions significantly improved IPH, IBN, LA, pigment content, fresh weight of adventitious roots, accumulations of aboveground biomass and underground biomass, root length, and root surface area in *S. jambos* (Figures 1, 2), in contrast to the pure planting (W). This result suggested that the mixed planting significantly improved the waterlogging adaptability of *S. jambos*. The increases in root length and root surface area expanded the contact area with soil, thereby facilitating the absorption of oxygen and nutrients (Ayi et al., 2016; Li et al., 2022). Moreover, *C. operculatus* has received few benefits from the mixed planting under waterlogging. The transformation of interspecific relationship under waterlogging condition may be attributed to the abundant adventitious roots of *C. operculatus*, which promoted the uptake and release of oxygen to water. This relationship improved the development and function of the adventitious root systems of *S. jambos* through aerobic rhizosphere. Therefore, the mixed planting pattern alleviated the negative responses of *S. jambos* to waterlogging. Bertness and Callaway (1994) and Lin et al. (2012) showed that the highest plant biomass accumulation and survival rates can be obtained under adverse external environmental stress, indicating the mutualistic relationship among adjacent plants under severe stressed conditions. Waterlogging-tolerant species can maintain high and stable photosynthetic capacity under submerged conditions. The chlorophyll is an essential component for the absorption and transmission of light energy and can adjust the ratio of light energy absorption and consumption in plants under stress and maximize the use of light energy (Klimov, 2003). Fv/Fm reflects the potential maximum photosynthetic capacity of plants and is an important indicator for studying photosynthesis. Although prolonged waterlogging reduced chlorophyll content, Pn, and Fv/Fm in both species as in previous studies (Bhusal et al., 2020), the mixed planting significantly increased pigment content, Pn, Gs, and Fv/Fm in *C. operculatus* and *S. jambos* under waterlogging stress (Tables 2 and 3; Figure 3). This finding suggested that mixed planting protected photosynthetic characteristics and was beneficial.

Waterlogging can trigger oxidative stress on plants at the sub-cellular level by overproducing ROSs and promoting their accumulation, such as hydrogen peroxide (H_2_O_2_) and $O_{2}^{\cdot -}$ . The levels of relative conductivity and MDA reflected cell membrane stability, which is an indicator of stress tolerance (Yang et al., 2011, 2015). As the final product of membrane lipid peroxidation, MDA content not only directly expresses the degree of damage in plants under waterlogging stress but also indirectly reflects the tolerance of plants to stress (Yang et al., 2011, 2015). Some stress-tolerant plants can develop complete antioxidant systems that protect cell structures and sub-cellular organelles from oxidative damage, including non-enzymatic antioxidant components, such as proline, soluble proteins, GSH, and ASA, and antioxidant enzymatic systems, including POD, SOD, and CAT. Both types of components can act as ROS scavengers and lipid peroxidation inhibitors (Yang et al., 2015; Miao et al., 2017). The high relative conductivity and MDA and $O_{2}^{\cdot -}$ levels (Figure 3) in *C. operculatus* and *S. jambos* under waterlogging stress suggested that waterlogging caused some oxidative stress and damaged the integrity of the cell membrane. However, their levels in the leaves of both plants decreased when the mixed planting was used, suggesting that the patter efficiently alleviated the damage due to waterlogging stress, especially in *S. jambos*. In addition, the mixed planting under waterlogging condition induced significant increases in the levels of GSH and soluble proteins in *S. jambos* and slightly increased SOD and CAT activities in *C. operculatus*, suggesting that *C. operculatus* and *S. jambos* employed different antioxidant systems to maintain the redox equilibrium and improve their waterlogging tolerance under waterlogging stress in the mixed planting.

The interactions of some hormones can activate defense responses and regulate plant growth and development under stress. IAA and GA_3_ play important roles in the regulation of plant growth and fruit development, and ABA and JA-Me can inhibit the growth-promoting effects of IAA and GA_3_ (Horbowicz et al., 2021). In the present study, the mixed planting under well-watered condition significantly increased the levels of IAA, ABA, GA_3_, and JA-Me in *C. operculatus* but induced significant decrease in IAA content and increase in JA-Me content in *S. jambos* (Figure 6), suggesting that mixed planting exerted a positive effect on *C. operculatus* but caused some negative effects on *S. jambos* under well-watered condition. Khan et al. (2018) suggested that waterlogging stress improves flooding tolerance of *Glycine max* by suppressing endogenous ABA production and increasing JA-Me content and the dynamics of these hormones play an important role in the regulation of photosynthesis. The mixed planting under waterlogging condition significantly increased the levels of GA_3_ and JA-Me in *C. operculatus* but induced significant decrease in their levels in *S. jambos* (Figure 6), indicating that *C. operculatus* benefited more in terms of growth advantages and waterlogging tolerance than *S. jambos* given that *S. jambos* gained more facilitation from the waterlogging mixed planting.

## Conclusion

In general, *C. operculatus* and *S. jambos* are waterlogging-tolerant species, but *C. operculatus* has a higher waterlogging tolerance than *S. jambos*. A competitive relationship was found between *C. operculatus* and *S. jambos* under water-watered condition, and *C. operculatus* showed better growth performance and gained more benefits. *S. jambos* suffered from some negative effects under the well-watered mixed planting. However, the competitive relationship under well-watered condition transformed into a mutualistic one under waterlogging condition, and the mixed planting significantly improved the waterlogging tolerance of *C. operculatus* and *S. jambos* in comparison with the monoculture under waterlogging condition, especially *S. jambos*. The results showed that the interspecific relationship between *C. operculatus* and *S. jambos* can be transformed by waterlogging stress and improve their waterlogging tolerance. This study shed light on the relegation activities in the riparian areas and ecological watershed, especially for suitable plant species selection and plant community construction.

## Data Availability Statement

The original contributions presented in the study are included in the article/Supplementary Material, further inquiries can be directed to the corresponding authors.

## Author Contributions

FY designed the experiment and rewrote the manuscript. JZ performed the experiments, analyzed the data, and wrote the draft manuscript. E-HC performed the revisions. D-DL, L-YG, and L-SX assisted to carry the partial experiments. L-FM provided funding and managed the experiment. All authors contributed to the article and approved the submitted version.

## Funding

This work was supported by Hainan Province Science and Technology Special Fund (ZDYF2022SHFZ054),Hainan Provincial Natural Science Foundation of China (Grant Nos. 421RC1033, 320RC507, and 421QN192), and National Science Foundation of China (Grant Nos. 32060240 and 31660165).

## Conflict of Interest

The authors declare that the research was conducted in the absence of any commercial or financial relationships that could be construed as a potential conflict of interest.

## Publisher’s Note

All claims expressed in this article are solely those of the authors and do not necessarily represent those of their affiliated organizations, or those of the publisher, the editors and the reviewers. Any product that may be evaluated in this article, or claim that may be made by its manufacturer, is not guaranteed or endorsed by the publisher.

## Supplementary Material

The Supplementary Material for this article can be found online at: https://www.frontiersin.org/articles/10.3389/fpls.2022.869418/full#supplementary-material
