# Supplementary figures and images for: Competitive Relationship Between Cleistocalyx operculatus and Syzygium jambos Under Well-Watered Conditions Transforms Into a Mutualistic Relationship Under Waterlogging Stress
Source: Front Plant Sci. 2022 Jun 10;13:869418. doi: 10.3389/fpls.2022.869418 (PMC9231832; doi:10.3389/fpls.2022.869418)

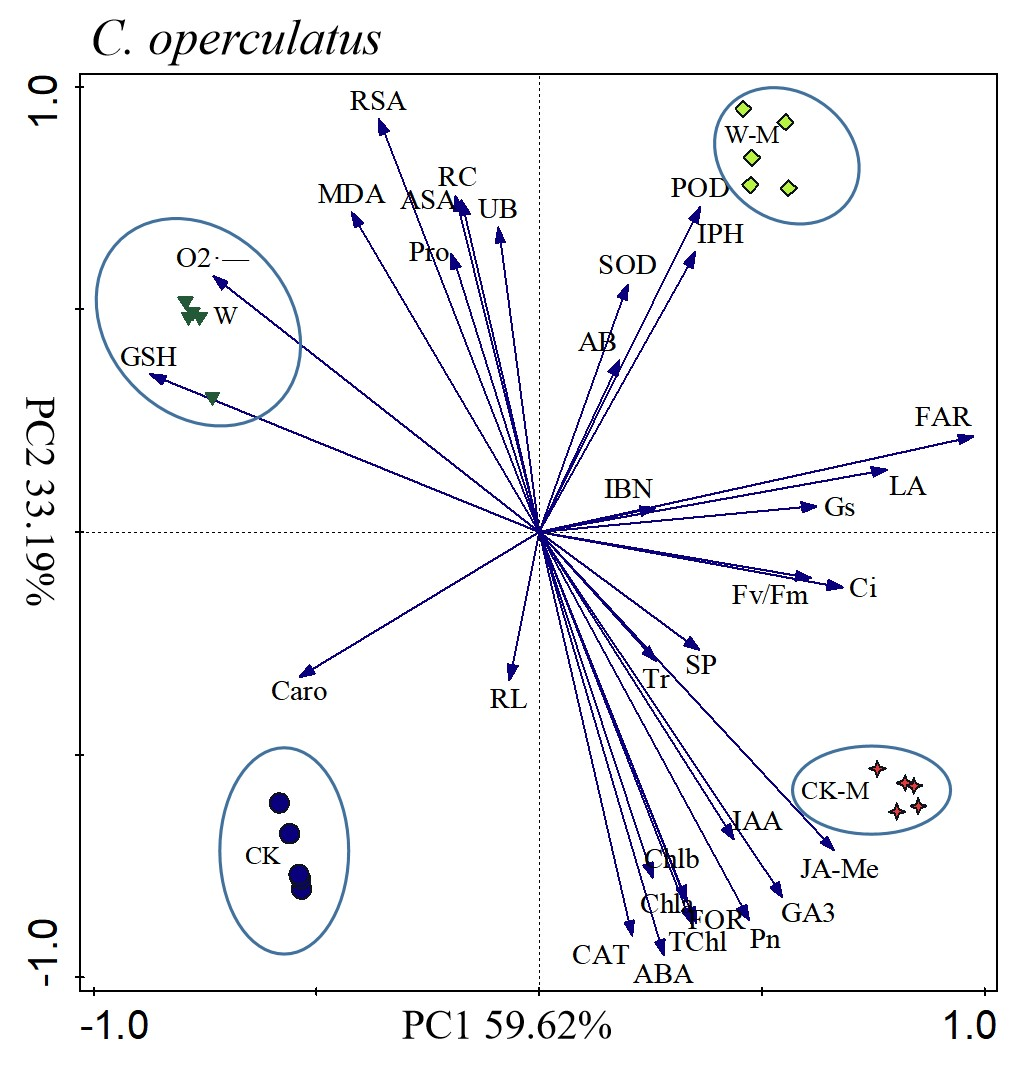

Supplement: Supplementary file 1 [file Image_1.TIF]

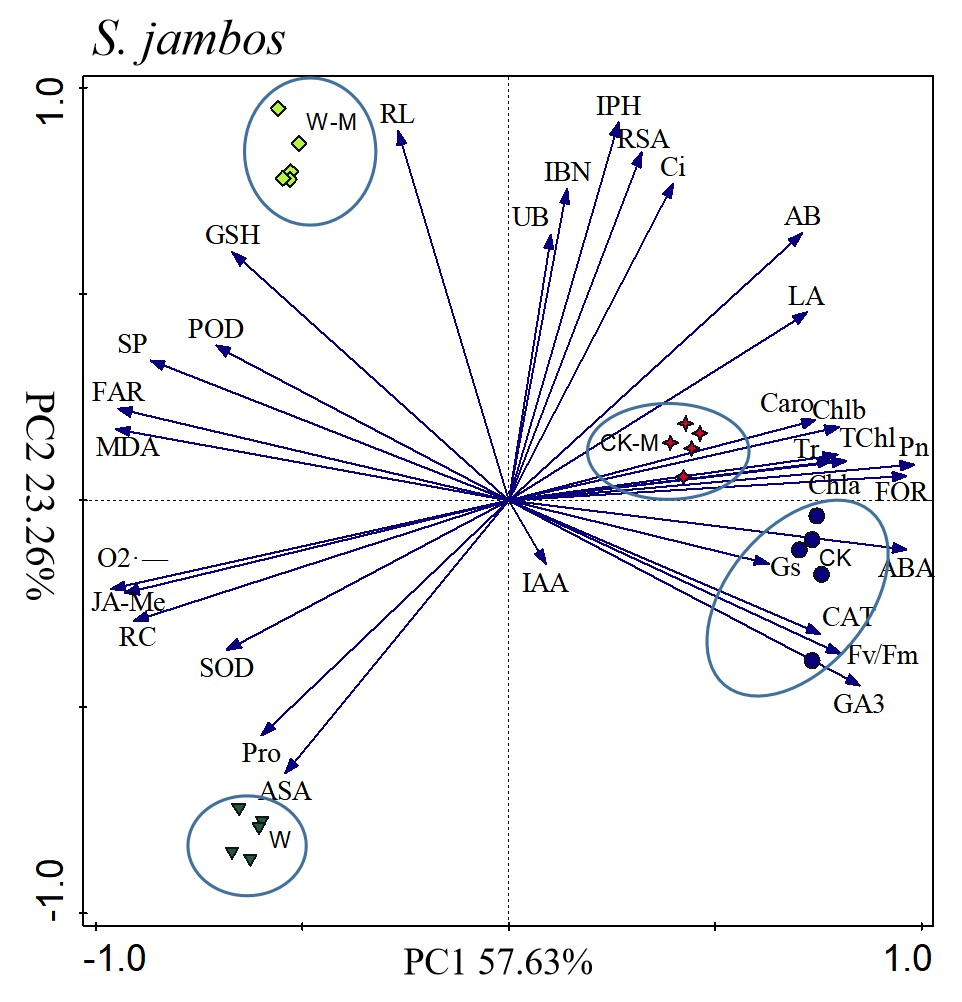

Supplement: Supplementary file 2 [file Image_2.TIF]
